# Regional variation in long-term care spending in Japan

**DOI:** 10.1186/s12889-022-14194-6

**Published:** 2022-09-23

**Authors:** Xueying Jin, Masao Iwagami, Nobuo Sakata, Takahiro Mori, Kazuaki Uda, Nanako Tamiya

**Affiliations:** 1 Department of Social Science, Center for Gerontology and Social Science, National Center for Geriatrics and Gerontology, Obu, Japan; 2 Health Services Research and Development Center, University of Tsukuba, Tsukuba, Japan; 3 Department of Health Services Research, Faculty of Medicine, University of Tsukuba, Tsukuba, Japan; 4 Heisei Medical Welfare Group Research Institute, Tokyo, Japan; 5 Department of General Internal Medicine, International University of Health and Welfare Narita Hospital, Narita, Japan

**Keywords:** Long-term care spending, Regional variation, Long-term care claims data

## Abstract

**Background:**

Health inequalities are widening in Japan, and thus, it is important to understand whether (and to what extent) there is a regional variation in long-term care (LTC) spending across municipalities. This study assesses regional variation in LTC spending and identifies the drivers of such variation.

**Methods:**

We conducted a cross-sectional study using publicly available municipality-level data across Japan in 2019, in which the unit of analysis was municipality. The outcome of interest was per-capita LTC spending, which was estimated by dividing total LTC spending in a municipality by the number of older adults (people aged ≥ 65). To further identify drivers of regional variation in LTC spending, we conducted linear regression of per-capita spending against a series of demand, supply, and structural factors. Shapley decomposition approach was used to highlight the contribution of each independent variable to the goodness of fit of the regression model.

**Results:**

In Fiscal 2019, per-capita LTC spending varied from 133.1 to 549.9 thousand yen (max/min ratio 4.1) across the 1460 municipalities analyzed, showing considerable regional variation. The included covariates explained 84.0% of the total variance in LTC spending, and demand-determined variance was remarkably high, which contributed more than 85.7% of the overall R^2^. Specifically, the highest contributing factor was the proportion of severe care-need level and care level certification rate.

**Conclusions:**

Our results demonstrate that, even after adjusting for different municipalities’ age and sex distribution, there is a large variation in LTC spending. Furthermore, our findings highlight that, to reduce the spending gap between municipalities, the issues underlying large variations in LTC spending across municipalities must be identified and addressed.

**Supplementary Information:**

The online version contains supplementary material available at 10.1186/s12889-022-14194-6.

## Background

Japan, the country with the oldest population, implemented a universal long-term care (LTC) insurance system in 2000. The Japanese LTC insurance system is one of the most comprehensive social care systems for older people in the world, built to assure fairness and efficient delivery of user-centered LTC services regardless of income. Japanese universal LTC system is highly decentralized, with municipality playing a key role in its operation. Municipalities operate as insurers, collect LTC insurance premiums, certify the need for LTC, provide insurance benefits and manage the LTC insurance finances. Regarding financing the LTC insurance, primary insured persons (aged 65 or over) and secondary insured people (40 to 64 years old) are contributing to 23% and 27% of total LTC budgets by paying insurance premiums. The other half of LTC budgets is covered by general tax (of which, 25% is covered by the national government, 12.5% is covered by prefectural governments and 12.5% is covered by municipal governments) [1, 2]. Therefore, fiscal and budgetary pressure on LTC expenditure varies across municipalities depending on their local needs.

Large regional variations in healthcare utilization and spending have been documented in many countries [3, 4]. Previous studies reported that demand factors such as demographics and health status largely explained regional variations [5, 6]. Supply factors such as density of physicians and competition were investigated, and the impact of these variables varied according to the structural factors [7]. Structural factors defined as political, economic, social, and organizational environments influenced regional variation [6, 7]. Evidence from the abovementioned studies is used to address the gap between regions and contribute to the financial sustainability of healthcare system. However, to our knowledge, whether (and to what extent) there is a regional variation in LTC spending across the municipalities remains unclear.

Therefore, this study aims to examine municipality-level variations in LTC spending and clarify the drivers of such variations using national-level LTC claims open data.

## Methods

### Japan’s LTC insurance system and services

Under the LTC insurance system, citizens aged 65 years or older, and those aged 40–64 years with health-related disabilities are eligible to receive LTC services including home, community, and facility-based care services. Eligibility for LTC is determined by municipalities according to nationally standardized assessments based on the extent of a physical or mental disability. Seven levels of long-term care-need certificates were established beginning with support levels 1 or 2, which are intended to provide preventive services. Care level 1 comprises users who are relatively less disabled, and care level 5 comprises users who are most disabled [8].

In principle, the insurer is the municipality where the person resides except in the following cases: when a person changes his/her residence by entering an LTC facility, the person is insured by the municipality where he/she lived before entering the facility. This domicile exception system was established to prevent municipalities that have a high concentration of LTC service providers from being the financially burdened [2].

### Data source and analysis unit

We used publicly available municipality data from opened LTC claims data 2019 (also known as Statistics of Long-term Care Benefit Expenditures), portal site of official statistics of Japan [9]. The unit of analysis in this study was a municipality in Japan. There are 47 prefectures in Japan, and each prefecture includes 15–179 municipalities. In total, there were 1724 municipalities in Japan as of the year 2019. Of these, we excluded municipalities that belong to wide-area unions due to lack of information on LTC spending because wide area unions are insurers of LTC instead of included municipalities. Additionally, we excluded municipalities whose population was smaller than 2000, because these municipalities were not allowed to publish based on the guidelines of the LTC claims database.

### Definitions of per-capita LTC spending

Per-capita LTC spending was calculated by dividing the total LTC cost in a municipality by the number of people aged ≥ 65 (who mostly use the LTC budgets) in that municipality. The expenditures are presented in Japanese thousand yen (equivalent to 9.1 US dollars or 7.8 Euros as of September 2021).

### Covariates

Among people aged ≥ 65 years, we further attempted to identify drivers of regional variation in LTC spending. Based on the literature review [5–7], possible predictors of regional LTC spending were grouped into three categories: demand, supply, and structural variables. The following variables, which are proxies for population attributes and health status, were selected as demand factors [7]: proportion by age group (65–84 and ≥ 85), proportion of females, proportion of severe care levels (care levels 3–5) among older adults, LTC certification rate (the proportion of older adults certified as requiring LTC),  per-capita medical (including inpatient and outpatient) cost and mortality. Supply factors refer to LTC resources and the delivery of services [6, 7]; therefore, we included the number of LTC facility beds per 1000 LTC beneficiaries, the number of LTC facility employees, and LTC provision (i.e., the proportion of LTC service users among those who are LTC certified) as variables. The data, pertaining to the number of LTC facility beds per 1000 LTC beneficiaries and the number of LTC facility employees, were for 2017; we used this information as a proxy since data for 2019 were unavailable. Structural covariates were the financial power index (i.e., standard financial revenues divided by amount of basic fiscal demand) and unemployment rate.

### Statistical analysis

Initially, we presented the distribution of per-capita LTC spending and covariates by calculating the coefficient of variation (CV) and max/min ratio. To reach a fairer comparison, we utilized age-sex adjusted LTC spending. To calculate this, an observed LTC spending was divided by its expected spending (O/E), and the O/E is multiplied by the mean of per-capita LTC spending in total municipalities. The expected spending was the predicted value of linear regression with per-capita LTC spending of each municipality as the dependent variable, vs. age and sex distribution as independent variables.

Then, we further performed multiple linear regression analyses to explore the drivers of municipal-level variation in LTC spending. Values of variance inflation factor that exceed 10 were considered to exhibit multicollinearity. Shapley approach was used to show the contribution of each independent variable to the overall R-square of linear regression [10]. Finally, a sensitivity analysis was performed to check if these results were applicable to people aged ≥ 40 (who are insured by LTC care system). The significance level was set at 5% and statistical analyses were performed using STATA ver. 16.

## Results

### Descriptive analysis

A total of 1460 municipalities were included in our final analysis after excluding the municipalities belonging to wide-area union (*n* = 219) and small municipalities (*n* = 45) whose population was under 2000. On average, the population comprised 51.3% females and 18.4% of the population were 85 years and older. Approximately 18.2% of older adults received LTC certification, and 86.6% received LTC services among the LTC beneficiaries (Table 1).

**Table 1:** Descriptive statistics for the demand, supply, and structural covariates (*n* = 1460)

	Mean	Sd	Min	Max	CV	Max/Min
Demand						
*Demography*						
Age groups						
65–84 years (%)	81.6	4.0	69.6	90.9	4.8	1.3
85 years or older (%)	18.4	4.0	9.1	30.4	21.6	3.4
Sex						
Female (%)	51.3	1.3	44.9	55.1	2.6	1.2
*Proxy of Health status*						
Care level certification rate (%)	18.2	2.8	10.6	27.8	15.1	2.6
Severe care level (%)	6.8	1.3	3.8	11.4	18.8	3.0
Per-capita medical cost (kJPY)	584.9	84.0	256.6	901.0	14.4	3.5
Per-capita inpatient cost (kJPY)	282.8	66.7	125.1	614.6	23.6	4.9
Per-capita outpatient cost (kJPY)	276.0	29.1	115.8	385.6	10.5	3.3
Mortality (per 1000 people)	14.0	4.4	3.4	40.2	31.2	11.8
Supply						
LTC provision rate among LTC beneficiaries (%)	86.6	7.8	56.7	133.9	9.0	2.4
Number of LTC facility beds per 1000 LTC beneficiaries	177.9	115.0	0	1797.8	-	64.6
Number of LTC facility employees per LTC beneficiaries	114.3	73.1	0	1217.8	-	64.0
Structural factors						
Financial capacity index	0.5	0.3	0.1	2.2	52.7	21.8
Unemployment rate (%)	4.0	1.1	0.8	10.5	28.6	13.8

Abbreviations: *LTC* Long-term care, *CV* Coefficient variation, *kJPY* Thousand yen

#### Crude and age-sex adjusted per-capita LTC spending

**Fig. 1:**
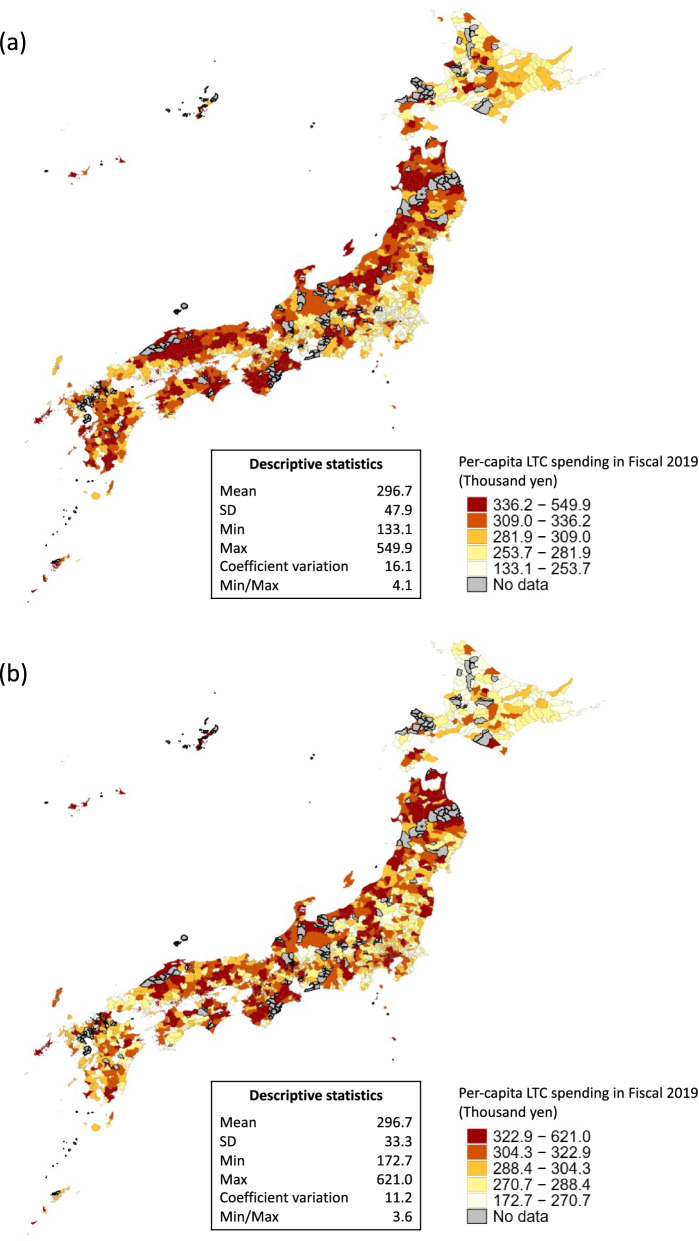
Unadjusted and age-sex adjusted per-capita LTC spending in municipalities. **A** Unadjusted LTC spending. **B** Age-sex adjusted LTC spending

The unadjusted per-capita LTC spending varied substantially across municipalities with a mean of 296.7 thousand yen (SD 47.9 k JPY), ranging from 133.1 to 549.9 thousand yen (max/min ratio 4.1), and showing a spending trend of “west high, east low”. However, following the adjustments for age and sex, the range of per-capita LTC spending reduced remarkedly, and the standard deviation dropped to 33.3 k JPY (Fig. 1).

#### Regional variation and predictors

Among people aged ≥ 65 years, the explained variance in the per-capita LTC spending was 84.0% in the regression model (Table 2). As shown in the Shapley-value variance indicating the decomposition of overall R^2^, demand factors explained the most of overall regional variation (85.7%), followed by supply factors (8.2%), and the structural factors (6.1%). More specifically, the proportion of severe care level and care level certification rate, and the proportion of people aged 85 years or older was the most contributing factor.

**Table 2:** Predictors of per-capita LTC spending for older people by municipalities: results of linear regression (*n* = 1460)

	Coefficient	95%CI	Shapley %R^2^
Demand			
85 years or older (%)	2.8	(2.3–3.3)	18.3
Female (%)	4.5	(3.5–5.6)	4.7
Care level certification rate (%)	7.2	(6.6–7.8)	22.8
Severe care level (%)	16.3	(15–17.6)	32.7
Older single-person households (%)	-1.0	(-1.3–-0.6)	4.3
Per-capita inpatient cost (kJPY)	-0.03	(-0.1–-0.01)	1.9
Per-capita outpatient cost (kJPY)	0.03	(-0.01–0.1)	1.0
Supply			
LTC provision rate among LTC beneficiaries (%)	1.8	(1.6–1.9)	7.9
Number of LTC facility beds per 1000 LTC beneficiaries	0.01	(-0.001–0.017)	0.3
Structure			
Financial capacity index	10.6	(5.1–16.1)	5.5
Unemployment rate (%)	2.0	(1–3)	0.6
Overall R^2^	0.840		

Abbreviations: *LTC* Long-term care, *kJPY* Thousand yen

The sensitivity analysis showed that regional variation in LTC spending was slightly higher in people aged ≥ 40 than in people aged ≥ 65 (Additional file 1), and drivers of variation were consistent in these two different groups. (Additional file 2).

## Discussion

This is the first study to examine variation in LTC spending across municipalities in Japan using national LTC claims open data and other municipality-level statistics. Per-capita LTC spending among older adults was more than four times higher in the highest-spending municipalities than in the lowest. After adjusting for demand, supply, and structural factors, 84.0% of the total variance in LTC spending was explained. Demand-determined variance was remarkably high, which contributed to 85.7% of the overall R^2^. The proportion of severe care level among older adults was the covariate that explained most of the regional variation in LTC spending.

Older adults contribute to a portion of total LTC spending by paying insurance premiums; therefore, older adults living in municipalities with higher per capita LTC spending also bear a higher financial burden. Our results showed a great variation in LTC spending among municipalities in Japan. Since regional variation explained by demographic differences is unavoidable, we also calculated age-sex adjusted per-capita LTC spending. Following this, regional variation reduced remarkably; however, there was still considerable variation in adjusted per-capita LTC spending across the municipalities.

The finding that demand factors largely explained regional variation in LTC spending is in line with previous studies from other developed countries. Van Noort and their colleagues reported that demand factors contributed to 55% of regional variation in the usage of in-home care in Netherlands [11]. Similar to LTC spending, demography and health explained 55–73% of regional variation in health care spending [6, 7, 12]. The care-need level certification rate explained a great deal of the regional variation in LTC spending, despite controlling for demographic and care-need level. As a possible explanation, supplier-induced demand in the LTC market may be related to a higher care-need level certification rate [13], because there was a strong correlation between care-need level certification rate and proportion of home care users. Thus, LTC beneficiaries living in municipalities that have an adequate supply of home care services can easily gain extensive information on these services and this may have been a link to higher care-need level certification rate. Another interpretation of this result is the health problems related to the care-need level certification rate. A Japanese study reported that a higher rate of patients (diseases of the circulatory system or cerebrovascular diseases) per 100,000 population is related to a higher care-need level certification rate [13]. Accordingly, efforts to prevent the onset and severity of lifestyle-related diseases may help reduce per-capita LTC spending.

Our results demonstrated that the proportion of severe care-need levels (care-need levels 3–5) among older adults contributes to approximately 32.7% of the overall R^2^. Therefore, to reduce the regional variations in LTC spending due to demand, a future study examining the factors associated with high care-need levels is needed. In addition, preventing the deterioration of the care level for mild and moderately disabled older adults may be linked to lower LTC spending. Previous studies have reported that in rehabilitation services [14], additional payments for case-specific care services [15] impact the deterioration of care level.

On the supply side, the number of LTC facilities per 1000 LTC beneficiaries explained 0.3% of the overall R^2^, and was positively associated with higher per-capita LTC spending. This association is consistent with previous studies, presenting a cost underestimation of home and community care since no benefits for informal care are captured in the Japanese LTC insurance system [16]. One Canadian study reported that home care is significantly less costly than residential care even when informal caregiver time is valued at replacement wage [17]. Thus, checking if there is an excessive provision of LTC facility services among municipalities may help reduce LTC expenditure. In addition, one possibility of admission to LTC facility may be that the family members may not be able to take care of seniors at home. The current Japanese LTC system can only provide insurance benefits in kind, including in-home services (e.g., home visits/day services and short-stay services/care) and services at facilities; and do not include cash benefits or other direct benefits for family caregivers. Studies are warranted to investigate whether additional in-home services (especially more sufficient short-stay services/care), as well as cash benefits or other direct benefits for family caregivers, could help older people with LTC needs stay at home if they want.

Of the structural factors, higher financial capacity indexes and unemployment rates were correlated with higher LTC spending. Municipalities with higher financial capacity indexes have more residents with higher incomes, leading to better access to LTC services and higher LTC spending [12, 15]. However, to locate and use LTC services, employees have to reduce their workload. Therefore they are less likely to access LTC services [12]. Likewise, the cost of taking time off of work is incurred by family members when looking for caregiver services.

Our study has several limitations. First, we used aggregate data at the municipality level; thus, caution is needed before applying our results to individuals to avoid ecological fallacy. Second, our study was not able to identify the uses of cross-municipal LTC services, which may have caused a bias in assessing the regional variations in LTC spending. Since many urban older adults enter LTC facilities in surrounding rural areas, the LTC spending is reimbursed by urban municipalities despite receiving services in rural areas. Therefore, the density of LTC facilities in a municipality—the supply—may be related to the needs of the surrounding urban areas. Third, the supply-driven factors are generally undesirable, and therefore, it is helpful to control for as many of these as possible. Care market competition (i.e., Herfindahl–Hirschman Index), labor (i.e., the density of nursing staff), and the average length of stay in LTC facilities may explain regional variations [6]; however, we could not adjust for these variables in this study. Fourth, we used care-need level as a proxy of health status; however, morbidity was not considered owing to data availability, even it is a sign of population health. Finally, the cross-sectional design cannot differentiate between cause and effect.

This study is the first attempt to examine variation in LTC spending using small area analysis. Since municipalities play a crucial role in LTC system in Japan, older adults in the same municipalities are more homogeneous in character than in larger areas such as prefecture. Consequently, our study displayed a wider variety of LTC spending across municipalities, making it easier to holistically identify and assess the issues of municipalities from the view of needs, supply, and structure. Regional variations could be a sign of inequity in access to LTC services and the inefficient and excessive use of LTC services [6]; however, we would like to stress that our study does not aim to quantify inefficiencies. We examined the relative importance of demand and supply factors as drivers of regional variations in LTC spending. Second, our study presented the extent to which predictors reduce regional variations. Furthermore, even after controlling for the age-sex distribution, there were considerable regional variations in LTC spending, and most were driven by the proportion of severe care levels among older adults. Thus, policies to reduce health disparities may be an effective way to reduce regional variations in LTC spending.

## Conclusions

In summary, we used national LTC claims open data, which cover all municipalities in Japan, to assess regional variation in LTC spending and identify its drivers. Our results revealed a large variation in LTC spending, despite adjusting for age and sex distribution across different municipalities. Adjusting for demand, supply, and system factors, 84.7% of the total variance in LTC spending was explained. Therefore, taking a closer look at municipalities from the demand, supply, and structural side is a necessary and effective way to reduce variation in LTC spending.

## Supplementary information

**Additional file 1.** Per-capita LTC spending in municipalities for people aged 40 and older (*n*=1460).

**Additional file 2.** Predictors of per-capita LTC spending for people aged 40 and older by municipalities: results of the linear regression analysis (*n*=1460).

## Data Availability

Data in this study are freely available on the following websites. *Long-term care insurance claims open data* (In Japanese) https://www.e-stat.go.jp/stat-search/files?page=1&toukei=00450351&tstat=000001031648*Observations of Municipalities* (In Japanese) https://www.e-stat.go.jp/stat-search?page=1&toukei=00200502

## Abbreviations

LTC: Long-term care
CV: Coefficient of variation
