# Supplementary figures and images for: Regional variation in long-term care spending in Japan
Source: BMC Public Health. 2022 Sep 23;22:1810. doi: 10.1186/s12889-022-14194-6 (PMC9508719; doi:10.1186/s12889-022-14194-6)

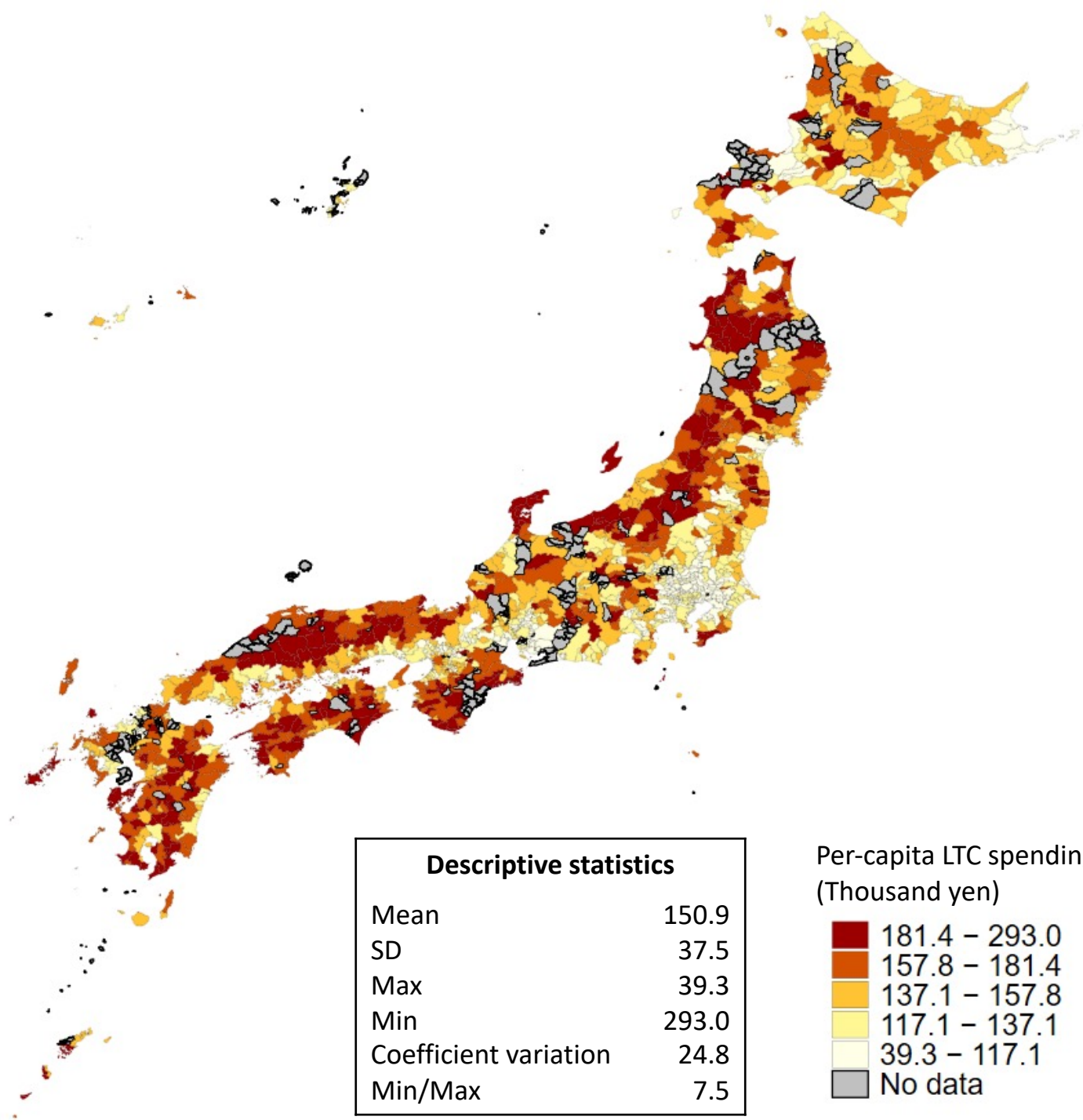

Supplement: Supplementary file 1 — Additional file 1. Per-capita LTC spending in municipalities for people aged 40 and older (n=1460). [file 12889_2022_14194_MOESM1_ESM.pdf]
