# Supplementary material for: Regional variation in long-term care spending in Japan
Source: BMC Public Health. 2022 Sep 23;22:1810. doi: 10.1186/s12889-022-14194-6 (PMC9508719; doi:10.1186/s12889-022-14194-6)
Supplement: Supplementary file 2 — Additional file 2. Predictors of per-capita LTC spending for people aged 40 and older by municipalities: results of the linear regression analysis (n=1460). [file 12889_2022_14194_MOESM2_ESM.docx]

Additional file 2. Predictors of per-capita LTC spending for people aged 40 and older by municipalities: results of the linear regression analysis (n=1460).

|  |  | Coefficient | 95%CI | Shapley %R2 |
| --- | --- | --- | --- | --- |
| Demand |  |  |  |  |
| 85 years or older (%) |  | 4.4 | (3.9 –4.8) | 27.0 |
| Female (%) |  | 2.3 | (1.7–2.8) | 5.9 |
| Care level certification rate (%) |  | 2.8 | (2.5–3.1) | 12.9 |
| Severe care level (%) |  | 18.3 | (17.1–19.6) | 31.9 |
| Per-capita Inpatient cost (kJPY) |  | -0.02 | (-0.03–-0.01) | 4.5 |
| Per-capita outpatient cost (kJPY) |  | 0.1 | (0.04–0.1) | 27.0 |
| Supply |  |  |  |  |
| LTC provision rate among LTC beneficiaries (%) |  | 0.8 | (0.7–0.9) | 2.9 |
| LTC facility users (%) |  | 0.005 | (-0.0003–0.01) | 0.7 |
| Structure | |  |  |  |
| Financial capacity index |  | 0.5 | (-2.6–3.6) | 12.3 |
| Unemployment rate (%) |  | 1.4 | (0.9–1.9) | 0.5 |
|  |  |  |  |  |
| Overall R^2^ |  | 0.924 |  |  |

Abbreviations: LTC=long-term care; kJPY=thousand yen.
